# Multi-omics joint analysis reveals the mechanism of flower color and fragrance variation in *Lilium cernuum*

**DOI:** 10.3389/fpls.2025.1489918

**Published:** 2025-04-28

**Authors:** Shaopeng Chen, Zhiqun Chen, Qianqian Zhuang, Hewen Chen

**Affiliations:** Jilin Agricultural Science and Technology University, College of Agriculture, Jilin, China

**Keywords:** *L. cernuum*, transcriptomics, metabolomics, volatile metabolomics, phenylpropanoid pathway

## Abstract

**Introduction:**

*Lilium cernuum*, a fragrant purple-red wild lily endemic to Northeast Asia, represents both ecological significance (as a key protected species) and horticultural value. While its white variant (*L. cernuum* var. *album*) exhibits distinct flower color and fragrance traits, the molecular mechanisms underlying these variations remain poorly understood. Previous studies attributed the low anthocyanin content in the white variant to LcMYB12 downregulation, yet comprehensive analyses of associated genes and metabolic pathways are lacking.

**Methods:**

This study employed integrated transcriptomics, metabolomics, and volatile metabolomics to systematically compare *L. cernuum* and its white variant. We analyzed differential gene expression in the phenylpropanoid and flavonoid biosynthesis pathways, quantified anthocyanin/flavonoid metabolites, and assessed volatile organic compound profiles.

**Results:**

The white variant showed significant reductions in flavonoids (catechin, epicatechin) and anthocyanins (cyanidin, pelargonidin, peonidin), linked to the downregulation of 58 genes in the flavonoid pathway—including *PAL*, *C4H*, *4CL*, and UFGT. Critically, UFGT suppression disrupted anthocyanin glycosylation, promoting degradation and vacuolar accumulation failure. Concurrently, phenylpropanoid pathway inhibition reduced p-coumaric acid synthesis, diminishing downstream anthocyanins and volatile compounds (eugenol/methyleugenol).

**Discussion:**

Our multi-omics approach reveals that flower color loss in *L. cernuum* var. album results from synergistic effects of transcriptional regulation and metabolic flux redirection. The UFGT-mediated glycosylation defect provides a novel explanation for anthocyanin instability in white petals. These findings complement prior genetic studies and establish a framework for targeted breeding of ornamental traits in Lilium species.

## 1 Introduction

Lily (*Lilium* spp.) is one of the world’s famous ornamental flowers, mainly used for cultivation and cut flowers. The ornamental characteristics of lilies are mainly in their floral organs, with lily flower colors including red, orange, white, and pink, but lacking blue and purple hues. *Lilium cernuum* belongs to the genus *Lilium*, specifically the Sect. Sinomartagon. This species is a key protected wild plant in China, with a narrow distribution and near-extinction status (Du et al., 2016). *L. cernuum* is distributed in northeastern China (Jilin Province, Liaoning Province), the Korean Peninsula, and the Russian Far East, with the Changbai Mountains (China, Korea) being the main distribution area of *L.cernuum*. It is the only fragrant purple-red wild lily species in northeastern China (Li et al., 2014; Du et al., 2016). According to Chung M Y, due to the altitude variation in the Changbai Mountains (known as Baekdu Mountain in Korea), plants in this area have been subject to glacial and interglacial cycles, resulting in a wide range of genetic variation among low to medium density populations, including different flower color variations in wild *L.cernuum* populations. The migration from south to north and the founder effect after the Last Glacial Maximum have significantly increased the genetic diversity of populations in southern Korea compared to those in northeastern China. *L. cernuum* growing in northeastern China or the Russian Far East is more adapted to cold climates (Chung et al., 2014), making it an important species for breeding Asian hybrid lilies (MacRae, 1998). Some studies suggest that the pink traits of Asian lilies may originate from *L. cernuum* (Yamagishi et al., 2014). Recent reports indicate that Yamagishi classified *L. cernuum* into two flower color types: the typical pink (*L. cernuum*) and the white variant (*L. cernuum* var. *album*). The anthocyanin content in the petals of the white variant is significantly low, possibly due to the downregulation of *LcMYB12* expression (Yamagishi, 2020). But it remains unclear whether other genes have changed, leading to the emergence of the white variant.

The main reasons for the diversity of flower color changes involve multiple factors and processes, including gene mutations and deletions, anthocyanin biosynthesis, pigment synthesis and degradation interactions, post-transcriptional regulation, hormone signaling pathways, the role of specific genes, and the regulation of fragrance compound synthesis. Different types and amounts of pigments (Wang and Yamagishi, 2019), including carotenoids, flavonoids, and alkaloids, are responsible for flower color formation (Ma et al., 2018; Zhu et al., 2019). While the purple, deep red, and pink color variations in lilies are caused by the varying content of flavonoids (anthocyanins). For example, purple-red Asian lily cultivars such as ‘Holean,’ ‘Monte Negro,’ and ‘Red Carpet’ are colored due to the presence of flavonoids, while white lilies lack anthocyanins or flavonol substances (Nørbæk and Kondo, 1999; Yamagishi et al., 2012).

Many studies have pointed out that the biosynthesis of anthocyanins in flowers is mainly regulated at the transcriptional level of its biosynthetic genes, and multiple transcription factors are involved in transcriptional regulation. In lilies, *LhR3MYB1* and *LhR3MYB2* have the ability to inhibit anthocyanin accumulation, with *LhR3MYB2* more strongly participating in negative regulation to limit anthocyanin accumulation (Sakai et al., 2019). In *Chrysanthemum×Morifolium*, *CmMYB4* and *CmMYB5* negatively regulate anthocyanin biosynthesis in chrysanthemums (Hong et al., 2019). Additionally, *MYB6* in *Populus tomentosa* (Wang et al., 2019) and *VvMYBA1* and *VvMYBA1-1* in *Vitis vinifera* (Xu et al., 2019). can promote the biosynthesis of anthocyanins and proanthocyanidins. The application of exogenous hormone ABA can upregulate *MdMYB110a* expression in *Malus pumila*, thereby increasing anthocyanin content in apple peels (Cho et al., 2020), suggesting that the *MYB* gene family and its subfamilies have both positive and negative regulatory roles in anthocyanin synthesis (Allan et al., 2008). However, the *MYB* gene alone cannot function; *MYB* regulation of anthocyanin synthesis requires the participation of *bHLH* and *WD40* to form the *MBW* complex to activate the promoters of anthocyanin synthesis structures (Jaakola, 2013). In *Freesia hybrida*, the *FhTTG1* gene in the *WD40* family synchronizes expression with proanthocyanidin and anthocyanin accumulation in *F. hybrida*, highly activating the promoters of genes related to anthocyanin biosynthesis (Shan et al., 2019). In the *Ziziphus jujuba* genome, 138 *bHLH* transcription factors were screened, with the *ZjTT8*, *ZjGL3a*, and *ZjGL3b* of subfamily III being highly correlated with anthocyanin synthesis (Shi et al., 2019). In the study of MBW complex regulation, in maize (Zea mays), the MYC transcription factor OsB2 can directly activate the expression of the MYB partner OsC1 gene, thereby promoting the expression of the WDR gene OsPAC1, forming the MBW complex and triggering the biosynthesis of anthocyanins. In Arabidopsis, the R2R3-MYB protein AtTT2 or AtPAP1 interacts with the WDR protein AtTTG1 to form the MW complex, which can activate the expression of its MYC partner AtTT8 gene. The accumulation of AtTT8 protein further forms the MBW complex, triggering the expression of structural genes (Bulanov et al., 2025).

Compared to the mechanism of flower color change, the mechanism of fragrance formation is less studied. Currently, the molecular mechanisms of fragrance production in some plants with significant aroma or special scents have been studied using transcriptomics and volatile compound profiling. The main volatile components of flower fragrance are a series of volatile organic compounds, collectively referred to as volatile organic compounds (VOCs). These compounds usually exist at low concentrations in plants and are released into the air through floral structures such as stomata and glands. The chemical composition of floral fragrance is complex and diverse, mainly including terpenes, aromatic compounds, aldehydes and ketones, esters, alcohols, and compounds containing sulfur and nitrogen (Ghissing and Mitra, 2022; Mostafa et al., 2022). Volatile analysis and RNA sequencing of four *Paeonia suffruticosa* species revealed 67 floral fragrance volatiles, with different compound contents in different *P. suffruticosa* varieties, and transcriptomics explained that 116 and 147 differentially expressed genes were related to terpene and benzene compound accumulation, respectively (Zhang et al., 2021). Analysis of floral scent compounds in four different flower colors of *Chimonanthus praecox* in Yunnan, China, identified 31 types of floral scent compounds, and principal component analysis showed that α-ocimene, Benzyl alcohol, Benzyl acetate, Cinnamyl acetate, Eugenol, and Indole were the main floral scent components distinguishing the four *C. praecox* flower colors in Yunnan (Meng et al., 2021). As an extremely important cut flower, lilies not only have beautiful and large flowers, but some lily varieties also have fragrance. Volatile substance analysis of five Oriental-trumpet hybrid lily varieties showed that the volatile composition of different types of hybrid lilies is not the same, with the floral volatile profiles of three Oriental-trumpet hybrids dominated by monoterpene hydrocarbons, monoterpene alcohols, and aldehydes, while the two Oriental hybrids were dominated by monoterpene alcohols, aldehydes, and phenylpropanoids, respectively (Johnson et al., 2016). As the only wild lily species in northeastern China with a strong and distinctive fragrance, *L. cernuum* has also been used in Asian lily hybridization. Yuka Inada introduced the fragrance trait of *L. cernuum* into Asian hybrid lilies by crossing *L. cernuum* twice with Asian hybrid lilies. Testing showed that the floral scent components released by *L. cernuum* were mainly benzene/phenylpropanoid compounds, monoterpenes, and fatty acids (Inada et al., 2023).

**Figure 1:**
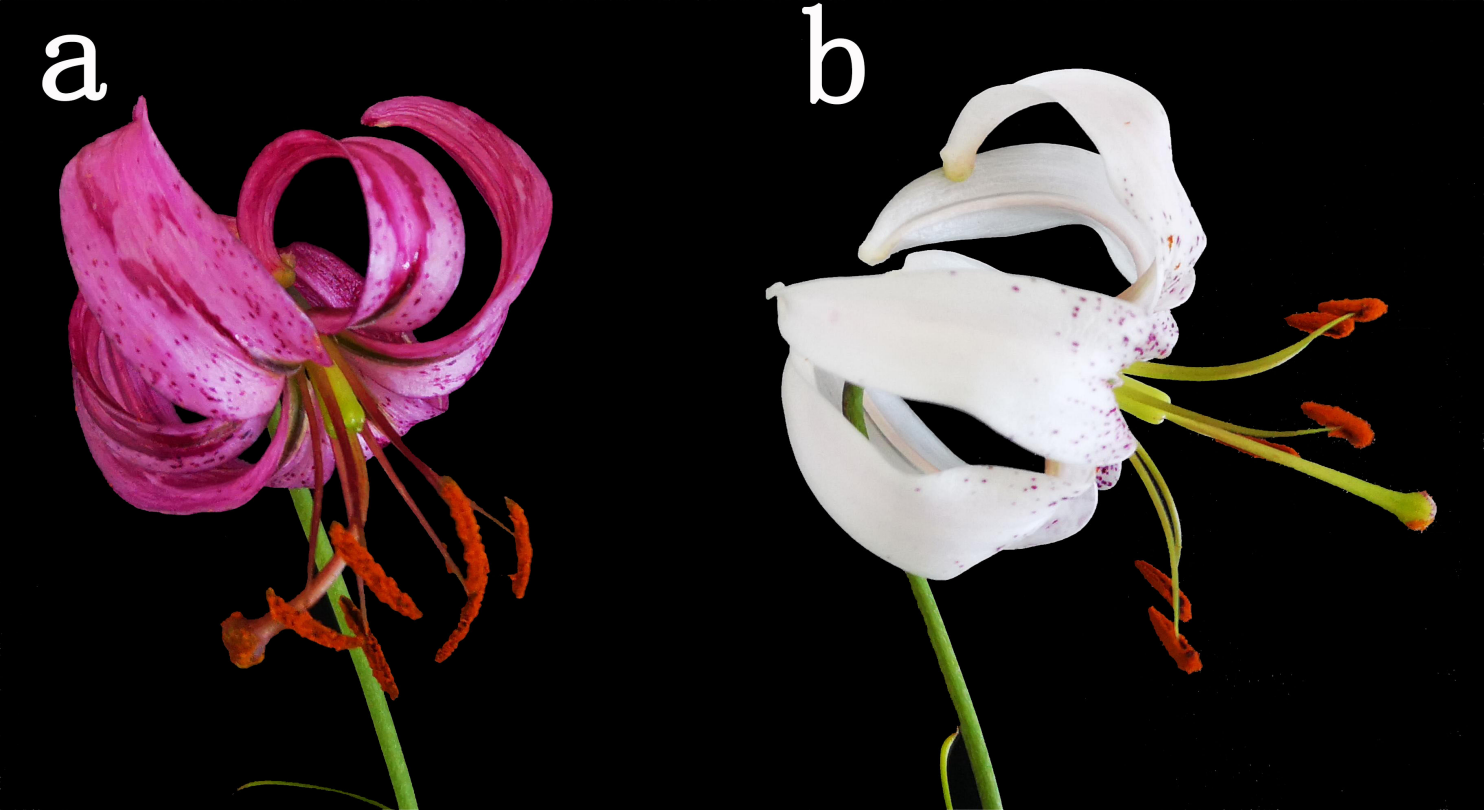
Flowers of *L. cernuum* **(a)** and *L. cernuum* var. *album* **(b)**.

*L. cernuum* is of great value in terms of plant genetic resources. In 2017, the research team discovered its white variant, *L. cernuum* var. *Album*, in the wild and successfully introduced it to the Horticulture Field of Jilin Agricultural Science and Technology College for cultivation (see **Figure 1**). The emergence of this white variant provides new material for studying the mechanisms of flower color formation in lilies. It is still unclear whether the variation in flower color affects the composition of fragrance volatiles. This study aims to explore the mechanisms of flower color variation in *L. cernuum* through the analysis of this color variant and to examine the changes in volatile compounds to investigate the linkage mechanism between flower color and fragrance variation. Through this research, we hope to reveal deeper connections between flower color and fragrance, thereby gaining a comprehensive understanding of *L. cernuum* and providing more theoretical support for the conservation and utilization of its genetic resources. The study of the genetic diversity and variation within *L. cernuum* holds substantial value for advancing genetic improvement and conservation strategies. By comprehensively analyzing the genetic background and biological traits of various flower color variants, this research offers fresh insights into the conservation of germplasm resources within the *Lilium* genus and provides a solid scientific foundation for genetic enhancement. This is particularly relevant for enhancing economically significant traits, such as flower color and fragrance, thereby increasing both the ornamental and commercial value of lilies. Furthermore, due to the overexploitation of wild resources and ongoing habitat destruction, species like *L. cernuum* face extinction risks. face a growing risk of extinction. Preserving its genetic diversity is therefore crucial. This study advances the understanding of the species’ genetic characteristics, providing a solid scientific foundation for developing effective conservation measures, safeguarding its genetic resources, and promoting sustainable development.

## 2 Experimental materials and methods

### 2.1 Materials and preparation

Robust and pest-free *L. cernuum* specimens, cultivated at the Horticulture Field of Jilin Agricultural Science and Technology College, served as the experimental subjects. These plants originated from Huashan Village, Linjiang City, Jilin Province (41°52’52.60’’N, 126°49’47.97’’E, altitude 567.5 m). The cultivation site is situated in a temperate continental monsoon climate within the northern temperate zone, characterized by distinct seasonal variations. The annual average temperature ranges from 3°C to 5°C. January, the coldest month, sees average temperatures of -18°C to -20°C, while July, the warmest month, experiences average temperatures of 21°C to 23°C. The annual temperature range averages 41.2°C. The growing season spans approximately 148.7 days per year, with an average frost-free period of 130 days. The site receives around 2,500 hours of sunlight annually and records average yearly precipitation between 650 mm and 750 mm. Freezing typically begins in October, with the frost period lasting 170 to 190 days. In June 2023, floral organs from two variants—*L. cernuum* (R) and *L. cernuum* var. *album* (W)—were collected. Samples were placed in pre-sterilized bags, with three parallel samples taken for each variant, totaling three replicates. After labeling and removing stamens and pistils, petals were immediately flash-frozen in liquid nitrogen and stored at -80°C upon returning to the laboratory.

### 2.2 Transcriptome database construction

#### 2.2.1 RNA extraction, verification, and sequencing

RNA was extracted from the two lily variants using ethanol precipitation combined with the CTAB-PVIOZOL method. The extracted RNA was dissolved in 50 µL of DEPC-treated sterile water. Quality and quantity assessments were performed using a Qubit fluorometer and a Qsep400 high-throughput bio-fragment analyzer. Qualified total RNA samples from *L. cernuum* (labeled as R-t) and *L. cernuum* var. *album* (labeled as W-t) underwent transcriptome sequencing, each with three biological replicates, resulting in six sample groups. Sequencing was outsourced to Wuhan Maiwei Metabolic Biotechnology Co., Ltd., utilizing the Illumina platform.

#### 2.2.2 Analysis and annotation of RNA-seq data

To ensure high-quality data, raw reads were filtered using fastp (v0.23.2) to remove adapter-containing sequences. Clean reads were assembled into transcripts using Trinity (v2.12.2), followed by clustering and de-redundancy with Corset (v1.09) (https://github.com/trinityrnaseq/trinityrnaseq). Coding sequences (CDS) were predicted from the Trinity-assembled transcripts using TransDecoder (TransDecoder5.3.0) (https://github.com/TransDecoder/), yielding corresponding amino acid sequences. De-redundant transcript sequences were annotated by aligning them with databases such as Nr (http://www.ncbi.nlm.nih.gov/), GO (http://www.geneontology.org), SwissProt (http://www.expasy.ch/sprot/), KEGG (http://www.genome.jp/kegg/), and EggNog (http://eggnogdb.embl.de/) using DIAMOND (v5.3.0). Transcription factors were predicted using iTAK software (Zheng et al., 2016).

#### 2.2.3 Identification of differentially expressed genes

Differential expression analysis between sample groups was conducted using DESeq2 to identify genes exhibiting significant differences in expression between the two biological conditions. To account for multiple hypothesis testing, the Benjamini-Hochberg method was applied to adjust the p-values, resulting in the calculation of the false discovery rate (FDR). Genes were then filtered based on the criteria of |log2 fold change| ≥ 1 and FDR < 0.05 to identify significantly differentially expressed genes.

#### 2.2.4 Quantitative real-time PCR analysis

qRT-PCR was employed to validate the transcriptional levels of differentially expressed genes (DEGs) in the petals of both lily variants. Reactions were performed using SYBR Premix Ex Taq (Tli RnaseH Plus, TaKaRa), with cDNA (diluted 20-fold) serving as the template. Protocols and reaction conditions adhered to the manufacturer’s instructions. Each qRT-PCR assay was conducted with three biological replicates, and relative gene expression levels were calculated using the 2^−ΔΔCT^ method. Subsequently, a one-way analysis of variance (ANOVA) was performed to compare gene expression differences across groups. To mitigate the risk of false positives from multiple comparisons, Tukey’s Honest Significant Difference (HSD) test was applied for pairwise comparisons, while the Benjamini-Hochberg method was utilized to control the false discovery rate (FDR). Gene-specific primers for qRT-PCR were designed using Primer Express (v3.0), with common lily actin (*LhActin*, NCBI accession number: JX826390) serving as the internal control. Details of the specific and internal control primers are listed in **Table 1**.

**Table 1:** Specific primers and internal reference primers.

Primer Names	Sequences(5’-3’)		Sequences(5’-3’)
Cluster-22099.11-F	TCATCGGATCATGGATAGTTC	R	CCAGTTGTTTCAAATGGGAG
Cluster-35249.4-F	TGGCACTGGCGAAGAATC	R	GCGAAAGCAGGGCACATA
Cluster-22099.14-F	TTTCATCGGATCATGGATA	R	TTCATTAGGGTCATTGGTTA
Cluster-27735.2-F	TCGTCCTCTACTACCGCATCT	R	ATCCCAACACCTCTTCCAG
Cluster-28792.0-F	GAGGGACTTGCCTTGGTGA	R	CTTGTTTCGGTTTCTGGAGTG
Cluster-31153.1-F	CGCTCTGTCAAGTCCGCTTTC	R	GTCGCTGGCGTCCATCTGT
Cluster-32178.3-F	GATAATCAAAGAGCAGGTCAAGC	R	GACAATGCCATCCCTACCG
Cluster-34110.8-F	CCTCCCAGTCATCCAGAAAG	R	TGTCATAGCCAATAACACCACC
Cluster-36742.5-F	GACAGACCCAAGATTCAGCA	R	GCATCTCCACTCGGAACACT
Cluster-22099.0-F	ACTCTGCCAGTGCTCTAACC	R	CACCATCAATTTCAGCGTCT
Cluster-17714.3-F	TCCTCCTGCGACCACCTTCC	R	CCTCCCTTCTTCCCGCCTGT
Cluster-22018.0-F	CAGCCAGACCATTCTCCCG	R	GCCAGCTTCAGCTCCACCT
Cluster-22018.3-F	GCATCCCAGACCATCCTCC	R	ATCCAGTATTGCCGAACCC
Cluster-24205.0-F	TCGCTTCTCGTCCACATTG	R	GTCCTCCTGCGTCTTCTTG
Cluster-25956.0-F	CCTACAACTGACTGGGCAACA	R	GGAAGGAGACTGGGTGAAGAG
Cluster-33883.13-F	GGTGGTCCTGCGATACTGG	R	CGACGGTCTCGACTGTGAG
Cluster-33883.15-F	CCGACTACTACTTCCGCATCA	R	GCTTTGGGACCTCGACAAC
Cluster-33883.25-F	TCCTCGCTGAGAACCCTAA	R	GCTGGTGGTGCAGAAGATG
Cluster-35293.2-F	GGTAAATGGAAGGGCAAGA	R	ATAGGAGTGGCAGCAGGGA
Cluster-35293.3-F	GGTAAATGGAAGGGCAAGA	R	GCAGCAGGGAAATGGATGG
Cluster-30144.2-F	TGCCAAGTTCTGAGCCCAATG	R	TCAAATGCGACACCAATCCC
Cluster-16648.0-F	CAAGTCCGAGAACTCCCTGAA	R	GATGCTGCCAAAGCTGATGT
Cluster-30144.1-F	TGCCAAGTTCTGAGCCCAATG	R	TCAAATGCGACACCAATCCC
Cluster-22672.0-F	CTCTGCCCACCTCAACACTG	R	TTTCGGTAACTCCTTGCTCAT
Cluster-30144.0-F	GACGACCTCCTGGACTTCAT	R	CATTGGGCTCAGAACTTGG
Cluster-30144.3-F	GACGACCTCCTGGACTTCAT	R	CATTGGGCTCAGAACTTGG
Cluster-33203.1-F	GACCATTCAGCCGCCAAGT	R	AAGCCACTCCATGTAACCACTCG
*LhActin*-F	GCATCACACCTTCTACAACG	R	GAAGAGCATAACCCTCATAGA

*LhActin*-F (R) denote the qRT-PCR primers for the internal reference gene.

### 2.3 Volatile metabolomics measurement

#### 2.3.1 Sample preparation and treatment

The lily petals stored in a -80°C freezer were taken out and ground into powder in liquid nitrogen. This process was performed for three biological replicates of each lily type, resulting in a total of six samples. The *L. cernuum* samples were labeled as R-GC, and the *L. cernuum* var. *album* samples were labeled as W-GC. 500 mg of the powder was immediately transferred to a 20 mL headspace vial (Agilent, Palo Alto, CA, USA) containing a NaCl saturated solution. The vials were sealed using crimp-top caps with TFE-silicone headspace septa (Agilent). For the SPME analysis, each vial was placed at 60°C for 5 minutes, then a 120 µm DVB/CWR/PDMS fiber (Agilent) was exposed to the headspace of the sample for 15 minutes at 60°C.

#### 2.3.2 GC-MS conditions

After sampling, desorption of the VOCs from the fiber coating was carried out in the injection port of the GC apparatus (Model 8890; Agilent) at 250°C for 5 minutes in splitless mode. The identification and quantification of VOCs were performed using an Agilent Model 8890 GC coupled with a 7000D mass spectrometer (Agilent), equipped with a 30 m × 0.25 mm × 0.25 μm DB-5MS (5% phenyl-polymethylsiloxane) capillary column. Helium was used as the carrier gas at a linear velocity of 1.2 mL/min. The injector temperature was maintained at 250°C. The oven temperature was programmed as follows: starting at 40°C (held for 3.5 minutes), increasing at 10°C/min to 100°C, at 7°C/min to 180°C, and at 25°C/min to 280°C, where it was held for 5 minutes. Mass spectra were recorded in electron impact (EI) ionization mode at 70 eV. The quadrupole mass detector, ion source, and transfer line temperatures were set to 150°C, 230°C, and 280°C, respectively. The MS was operated in selected ion monitoring (SIM) mode for the identification and quantification of analytes.

#### 2.3.3 Differential metabolite screening

Differential metabolites between *L. cernuum* and *L. cernuum* var. *album* were initially identified based on the Variable Importance in Projection (VIP) scores derived from the OPLS-DA model. These metabolites were then further refined using the false discovery rate (FDR) obtained from univariate analysis. The screening criteria for significant differences were defined as VIP > 1 and either a fold change ≥ 2 or ≤ 0.5.

### 2.4 Broad-spectrum targeted metabolomics measurement

#### 2.4.1 Dry sample extraction

The lily petals stored in a -80°C freezer were taken out and ground into powder in liquid nitrogen. This procedure was performed for three biological replicates of each lily type, resulting in a total of six samples. The *L. cernuum* samples were labeled as R-MS, and the *L. cernuum* var. *album* samples were labeled as W-MS. Using vacuum freeze-drying technology, the biological samples were placed in a lyophilizer (Scientz-100F) and then ground (30 Hz, 1.5 minutes) into powder using a grinder (MM 400, Retsch). Next, 50 mg of sample powder was weighed using an electronic balance (MS105DM) and 1200 μL of -20°C pre-cooled 70% methanolic aqueous internal standard extract was added (for samples weighing less than 50 mg, the extract was added at a rate of 1200 μL per 50 mg sample). The mixture was vortexed for 30 seconds every 30 minutes, for a total of six times. After centrifugation (12,000 rpm, 3 minutes), the supernatant was aspirated, filtered through a microporous membrane (0.22 μm pore size), and stored in an injection vial for UPLC-MS/MS analysis.

#### 2.4.2 UPLC conditions

The sample extracts were analyzed using a UPLC-ESI-MS/MS system (UPLC, ExionLC™ AD, https://sciex.com.cn/) coupled with a tandem mass spectrometry system (https://sciex.com.cn/). The analytical conditions were as follows: UPLC column, Agilent SB-C18 (1.8 µm, 2.1 mm × 100 mm); the mobile phase consisted of solvent A (pure water with 0.1% formic acid) and solvent B (acetonitrile with 0.1% formic acid). Sample measurements were performed using a gradient program starting at 95% A and 5% B. Over 9 minutes, a linear gradient to 5% A and 95% B was programmed, and this composition was maintained for 1 minute. Subsequently, the composition was adjusted to 95% A and 5% B within 1.1 minutes and held for 2.9 minutes. The flow rate was set to 0.35 mL/min; the column oven was set to 40°C; the injection volume was 2 μL. The effluent was alternately connected to an ESI-triple quadrupole-linear ion trap (QTRAP)-MS.

#### 2.4.3 ESI-Q TRAP-MS/MS

The ESI source operation parameters were as follows: source temperature 550°C; ion spray voltage (IS) 5500 V (positive ion mode)/-4500 V (negative ion mode); ion source gas I (GSI), gas II (GSII), and curtain gas (CUR) were set at 50, 60, and 25 psi, respectively; and the collision-activated dissociation (CAD) was set to high. QQQ scans were acquired as MRM experiments with collision gas (nitrogen) set to medium. The DP (declustering potential) and CE (collision energy) for individual MRM transitions were optimized. A specific set of MRM transitions was monitored for each period according to the metabolites eluted during that period.

The ESI source operation parameters were as follows: source temperature 550°C; ion spray voltage (IS) 5500 V (positive ion mode)/-4500 V (negative ion mode); ion source gas I (GSI), gas II(GSII), curtain gas (CUR) were set at 50, 60, and 25 psi, respectively; the collision-activated dissociation(CAD) was high. QQQ scans were acquired as MRM experiments with collision gas (nitrogen) set to medium. DP(declustering potential) and CE(collision energy) for individual MRM transitions was done with further DP and CE optimization. A specific set of MRM transitions were monitored for each period according to the metabolites eluted within this period.

#### 2.4.4 Differential metabolite screening

The procedure for differential metabolite screening was identical to that outlined in Section 2.3.3.

### 2.5 Analysis of Volatile Metabolomics and Broad-Spectrum Targeted Metabolomics

#### 2.5.1 PCA

Unsupervised PCA (principal component analysis) was performed using the ‘prcomp’ statistics function within R (www.r-project.org). The data was unit variance scaled before performing the unsupervised PCA.

#### 2.5.2 Hierarchical cluster analysis and pearson correlation coefficients

The HCA (hierarchical cluster analysis) results of samples and metabolites were presented as heatmaps with dendrograms, while Pearson correlation coefficients (PCC) between samples were calculated using the ‘cor’ function in R and presented as heatmaps only. Both HCA and PCC were performed using the R package ‘ComplexHeatmap’. For HCA, normalized signal intensities of metabolites (unit variance scaling) were visualized as a color spectrum.

#### 2.5.3 Differential metabolites selection

For two-group analysis, differential metabolites were determined by VIP (VIP > 1) and absolute Log2FC (|Log2FC| ≥ 1.0). VIP values were extracted from OPLS-DA results, which also contained score plots and permutation plots, generated using the R package ‘MetaboAnalystR’. The data was log-transformed (log2) and mean-centered before OPLS-DA. To avoid overfitting, a permutation test (200 permutations) was performed.

#### 2.5.4 KEGG annotation and enrichment analysis

Identified metabolites were annotated using the KEGG Compound database (http://www.kegg.jp/kegg/compound/), and annotated metabolites were then mapped to the KEGG Pathway database (http://www.kegg.jp/kegg/pathway.html). Pathways with significantly regulated metabolites were then fed into MSEA (metabolite sets enrichment analysis), and their significance was determined by hypergeometric test p-values.

## 3 Results and analysis

### 3.1 Transcriptomic analysis

#### 3.1.1 Sequencing quality and differential gene screening

**Figure 2:**
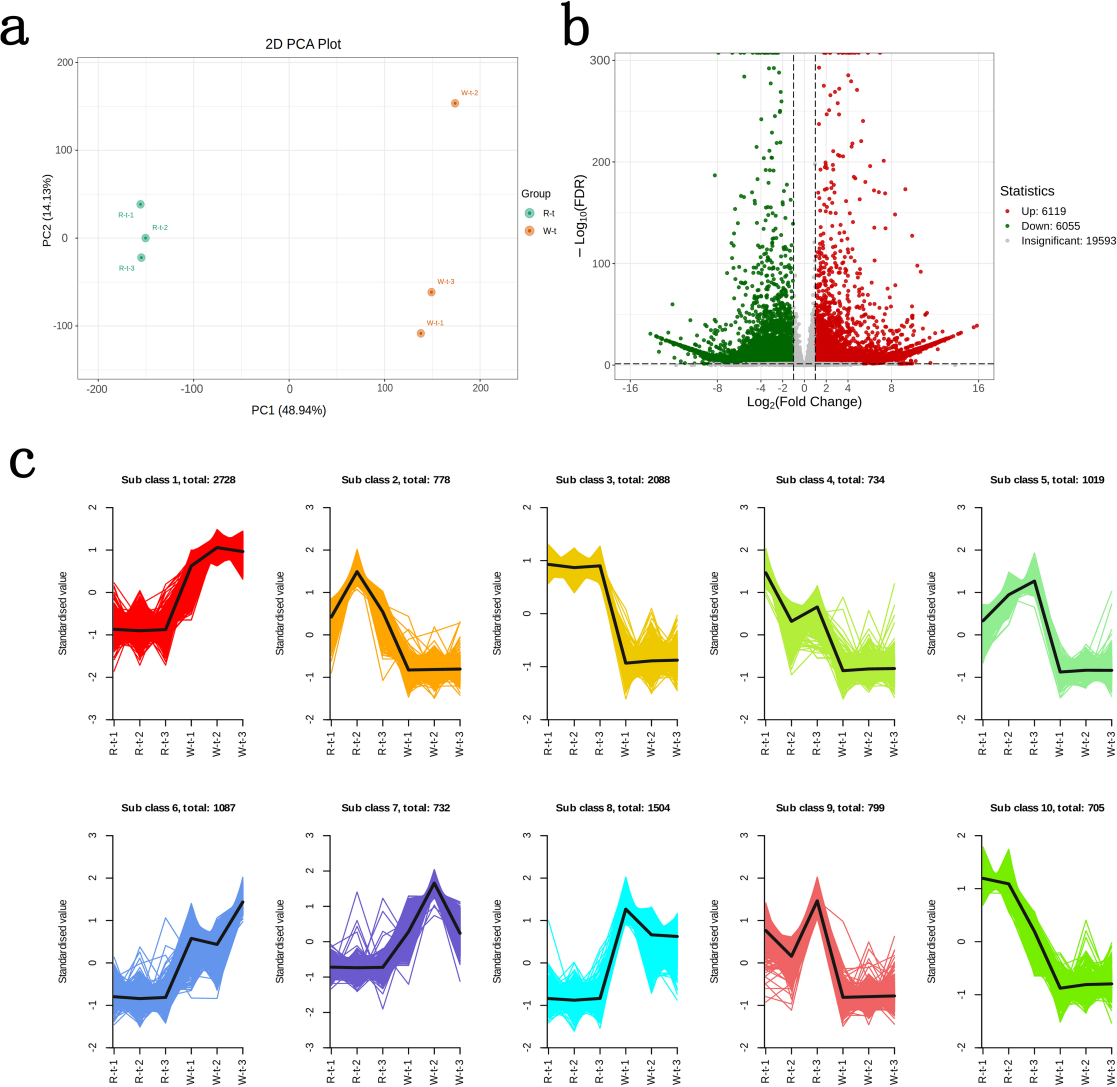
Differential gene screening and expression clustering analysis. **(a)** PCA plot of R-t and W-t. **(b)** Volcano plot of differential genes. **(c)** K-means clustering analysis of differential genes.The horizontal axis represents the samples, and the vertical axis represents the normalized expression levels.

Through transcriptomic analysis of the petals of two lily color varieties, R-t generated 46,676,986 clean reads, and W-t generated 42,606,872 clean reads. The error rate was kept below 0.02%. The Q30 values ranged between 94.70% and 95.31%, and the GC content ranged between 49.47% and 50.60%. The PCA plot showed that PC1 and PC2 contributed 63.07%, with the two lily varieties clearly separated (**Figure 2a**). The annotation rate reached 73.81% after comparing the reads with six databases: KEGG, NR, SwissProt, KOG, GO, and Pfam, indicating that the transcriptomic analysis is reliable and can be used for subsequent analysis. Differential gene screening of the obtained clean reads revealed 12,174 differentially expressed genes (DEGs) between R-t and W-t, with 6,055 genes downregulated and 6,119 genes upregulated (**Figure 2b**). According to K-means clustering analysis, the DEGs were divided into 10 subclasses. Compared to R-t, W-t showed higher gene expression levels in subclasses 1, 6, 7, and 8, while in subclasses 2, 3, 4, 5, 9, and 10, W-t exhibited lower gene expression levels compared to R-t (**Figure 2c**). These genes may play a crucial role in flower color change.

#### 3.1.2 GO annotation classification and KEGG analysis

**Figure 3:**
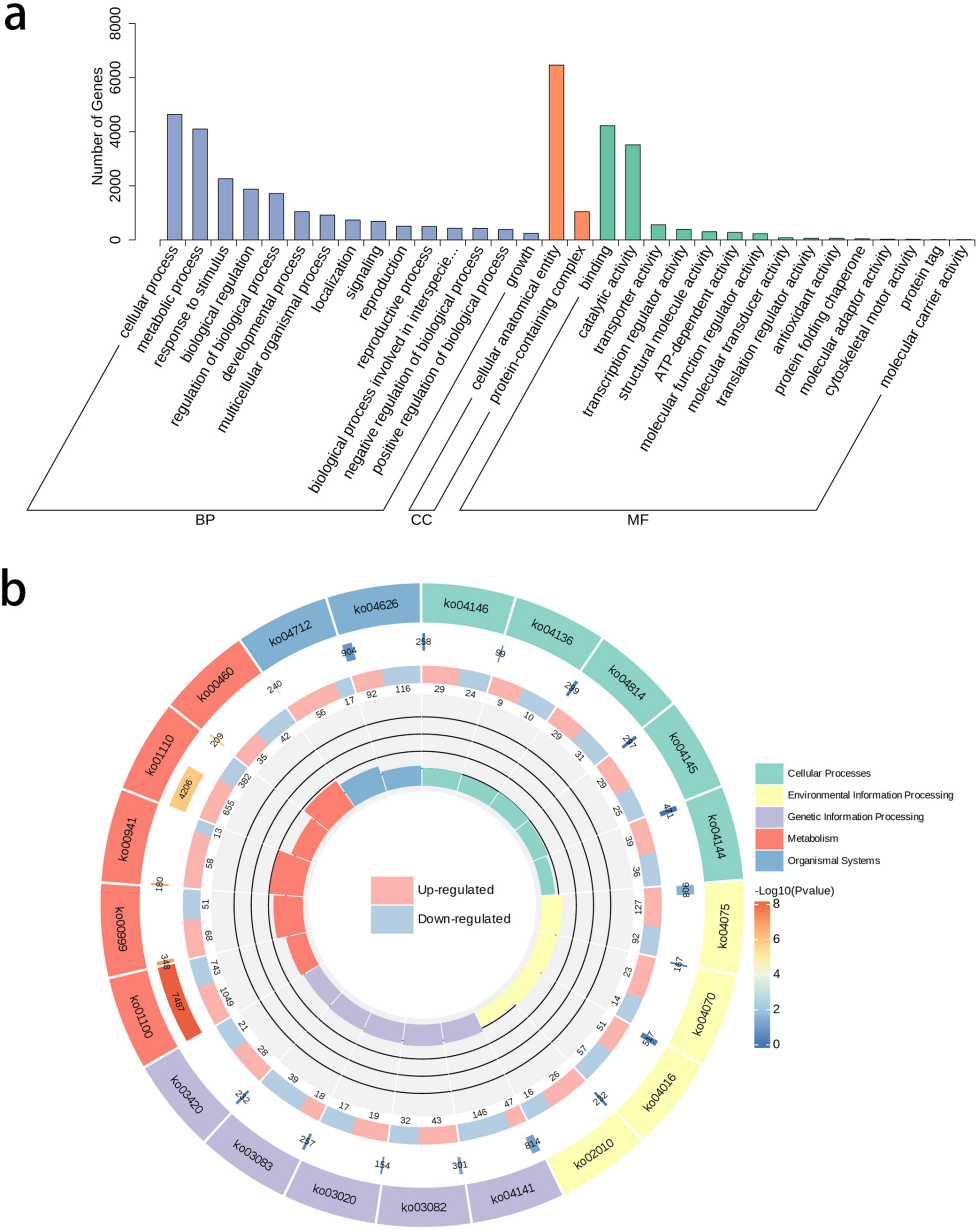
GO annotation classification statistics and KEGG analysis. **(a)** The horizontal axis represents the secondary GO terms, and the vertical axis shows the number of differential genes in each GO term. **(b)** From outer to inner, the first ring represents the KEGG pathway, with different colors representing different KEGG classifications; the second ring shows the number of genes in the background within this classification and the q-value. The longer the bar, the more genes there are, and the redder it is, the more significant the enrichment; the third ring shows the proportion of upregulated and downregulated genes, with light red representing the proportion of upregulated genes and light blue representing the proportion of downregulated genes; specific values are shown below; the fourth ring shows the RichFactor values for each classification, with each small grid of the background auxiliary line representing 0.2.

The GO annotation statistics of differential genes revealed several key biological processes and molecular functions related to flower color variation during pigment biosynthesis. In the Biological Process (BP) category, differential genes were mainly concentrated in Cellular and Metabolic Processes. The Cellular Component (CC) category data indicated significant changes in molecular components within organelles related to pigment synthesis and storage, possibly implying regulation of plastid formation or function in petals. In the Molecular Function (MF) category, genes involved in catalytic activation and protein binding were highly enriched, suggesting their potential roles in regulating anthocyanin biosynthesis and transport (**Figure 3a**). The KEGG annotation of differential genes showed distinct differential expressions in multiple metabolic pathways. These differential genes were mainly concentrated in Flavonoid biosynthesis (Ko00941), Biosynthesis of secondary metabolites (Ko01110), and Metabolic pathways (Ko01100) in the Metabolism process. Among these, the RichFactor value was the highest in the Flavonoid biosynthesis pathway. In this pathway, 58 differential genes were upregulated and 13 were downregulated in R-t compared to W-t (**Figure 3b**). In flavonoid biosynthesis, these differentially expressed genes mainly belong to the transferase family, reductase family, synthetase family, isomerase family, and oxidoreductase family. Key genes include flavanone 3-hydroxylase and chalcone synthase. Among them, genes related to the chalcone and stilbene synthases, N-terminal domain account for 26.8%, playing a crucial role in the biosynthesis of flavonoids and anthocyanin pigments.

### 3.2 Differential analysis of petals volatile organic compounds and petal metabolomics

#### 3.2.1 Differential analysis of petals volatile organic compounds

**Figure 4:**
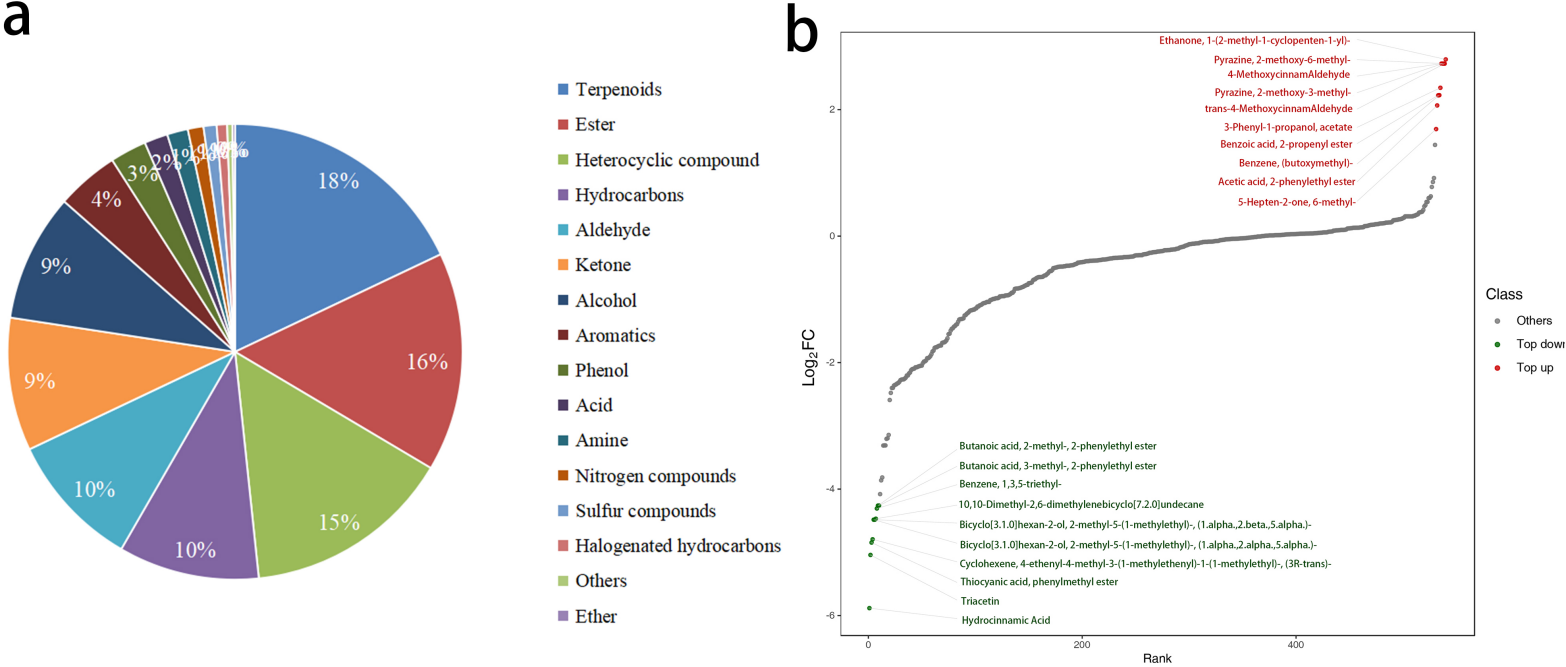
Differential analysis of VOCs between W-GC and R-GC. **(a)** Composition ratio of metabolites. **(b)** The horizontal axis represents the cumulative number of substances arranged in order of fold change from small to large, and the vertical axis represents the logarithmic value of fold change with a base of 2. Each point represents a substance, with green points representing the top 10 downregulated substances and red points representing the top 10 upregulated substances.

This study performed an overlay analysis of the total ion chromatograms (TIC) obtained from mass spectrometry detection of various quality control samples. The results showed a high degree of overlap in the TIC curves, with consistent retention times and peak intensities. This consistency indicates that the mass spectrometry signals remained stable across different detection times for the same sample, confirming their reliability for subsequent analysis. A total of 540 VOCs were detected in the petals, which were classified into 16 categories. Among these, terpenoids were the most abundant, with 97 types accounting for 18% of the total. This was followed by esters, with 84 types accounting for 15.6%, and heterocyclic compounds, with 80 types accounting for 14.8%. Together, these three classes of compounds accounted for 48.4% of the total (**Figure 4a**). Comparing the fold changes in VOCs quantification between W-GC and R-GC, 120 of the 540 VOCs showed changes. Compared to R-GC, 110 VOCs were significantly downregulated in W-GC, while only 10 VOCs were significantly upregulated (**Figure 4b**).

#### 3.2.2 KEGG functional annotation and enrichment analysis of petal VOCs

**Figure 5:**
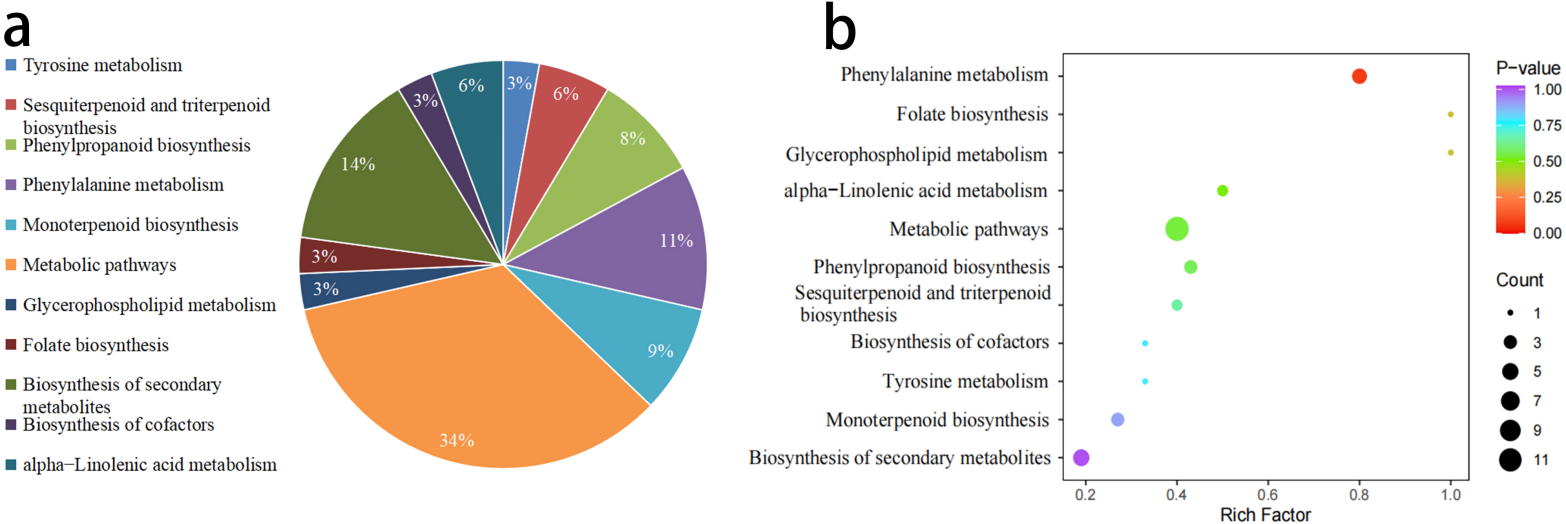
Comparison and enrichment of VOCs Pathways between W-GC and R-GC. **(a)** The proportion in the pie chart represents the ratio of differential metabolites annotated to that pathway out of the total annotated differential metabolites. **(b)** The horizontal axis indicates the Rich Factor for each pathway, and the vertical axis lists the pathway names (sorted by P-value). The color of the points reflects the P-value, with redder points indicating more significant enrichment, and the size of the points representing the number of enriched differential metabolites.

KEGG annotation was performed on the 120 VOCs, and 21 of these VOCs were annotated to 11 metabolic pathways. The classification of these results is shown in **Figure 5**. Among the annotated VOCs, 12 were concentrated in the Metabolic pathways process, accounting for 57.14%, followed by the Biosynthesis of secondary metabolites process, which included 5 significantly different VOCs, accounting for 23.81% (**Figure 5a**). Based on the results of the differential metabolites, KEGG pathway enrichment analysis was performed. The metabolic pathways were ranked and displayed according to their P-value, from smallest to largest across the 11 metabolic processes (**Figure 5b**). The results indicated that in the Phenylpropanoid biosynthesis process, the enrichment index of volatile differential metabolites was relatively high, and the difference was extremely significant.

#### 3.2.3 Sensory flavor characterization of differential VOCs in petals

**Figure 6:**
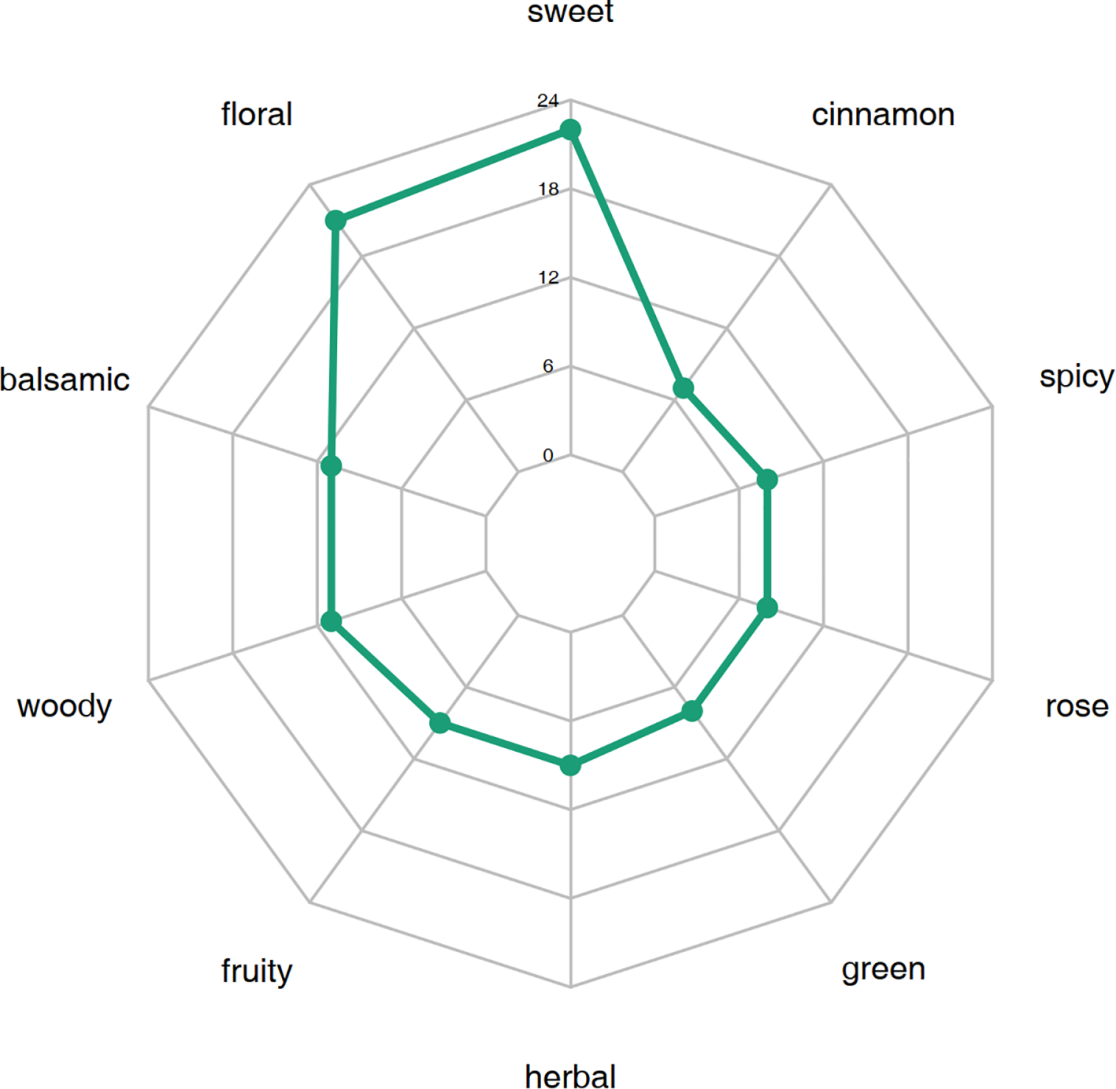
Sensory flavor characterization analysis of differential VOCs in petals.

By comparing the differential metabolites identified based on the screening criteria in W-GC and R-GC and their annotated sensory flavor characteristics, a radar chart was drawn for the top 10 sensory flavors with the highest number of annotations (**Figure 6**). The results showed that the differential VOCs between W-GC and R-GC were mainly concentrated in sweetness and floral aroma. In sweetness, 22 VOCs were annotated, including Acetophenone, 4’-hydroxy-, BenzAldehyde, BenzAldehyde, 2-methyl-, Hydrocinnamic Acid and Methyleugenol, with five VOCs being annotated to the Metabolic pathways, Phenylalanine metabolism, and Phenylpropanoid biosynthesis pathways, all of which were reduced in content. In floral aroma, 21 VOCs were annotated, including BenzeneacetAldehyde, Benzeneacetic acid, Eugenol, and trans-Isoeugenol, with four VOCs also annotated to the Metabolic pathways, Phenylalanine metabolism, and Phenylpropanoid biosynthesis pathways, all of which were also reduced in content. This suggests that during the transition of *L. cernuum* from red to white, some of the sweetness and floral aroma characteristics change.

The outermost circle names represent sensory flavor characteristics. The numbers corresponding to the green points indicate the number of occurrences of each sensory flavor characteristic, i.e., the number of differential metabolites annotated to that sensory flavor characteristic.

#### 3.2.4 Differential metabolite analysis of petals

**Figure 7:**
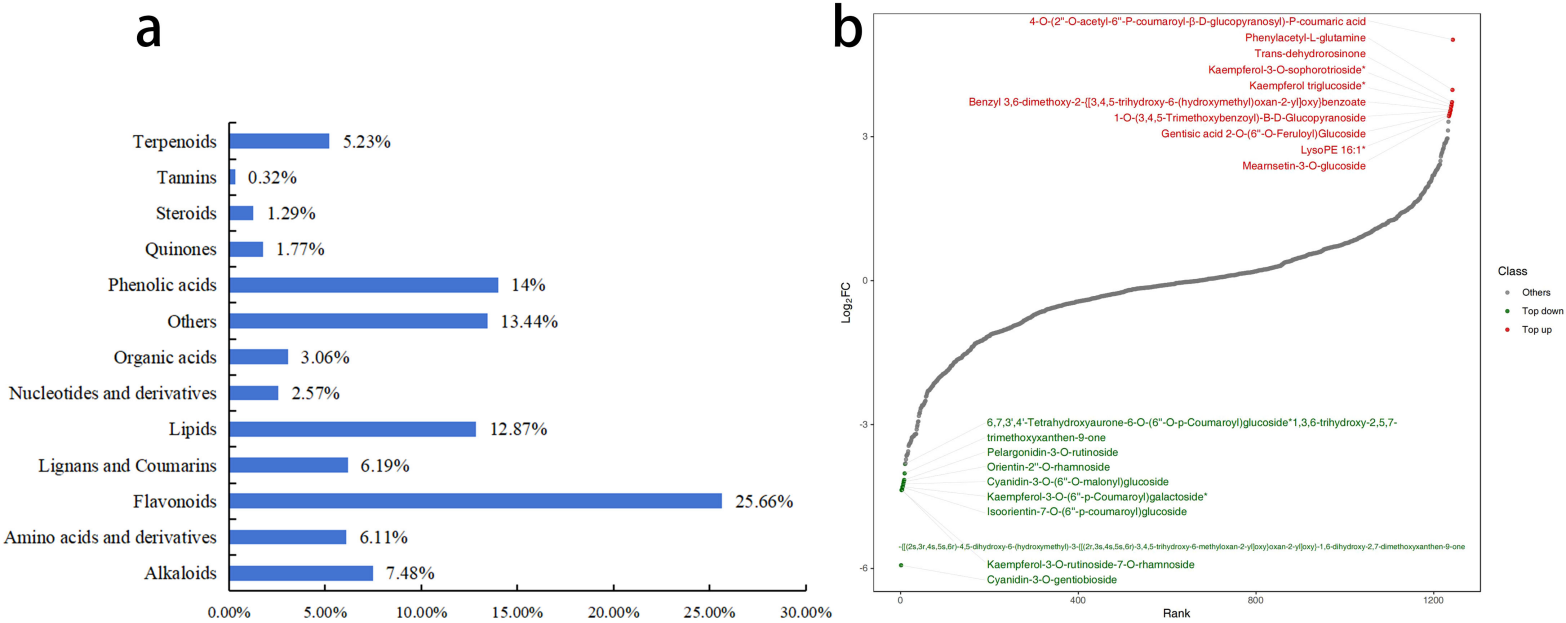
Differential analysis of metabolites between R-MS and W-MS. **(a)** Composition proportion of metabolites. **(b)** The horizontal axis represents the cumulative number of substances ranked by fold change from small to large, and the vertical axis represents the logarithm of the fold change with a base of 2. Each point represents a substance, with green points representing the top 10 down-regulated substances and red points representing the top 10 up-regulated substances.

The overlay analysis of the TIC revealed a high degree of overlap in the curves for metabolite detection, with consistent retention times and peak intensities. This demonstrates excellent signal stability, confirming that the data is reliable and suitable for subsequent analysis. A total of 1,243 metabolites were detected in the petals, which were classified into 13 categories. Among these, Flavonoids were the most abundant, with 319 metabolites accounting for 25.7%, followed by Phenolic acids with 174 metabolites, accounting for 14.0% (**Figure 7a**). Comparing the fold change in metabolite quantification between R-MS and W-MS, there were 419 significantly different metabolites (DMs) among the 1,243 metabolites in the petals. Compared to R-MS, W-MS had 235 metabolites that were significantly down-regulated, of which 119 were Flavonoids, accounting for 50.64%. On the other hand, 184 metabolites were significantly up-regulated (**Figure 7b**), among which Lipids were the most abundant, reaching 50 metabolites, accounting for 42.02%.

#### 3.2.5 KEGG functional annotation and enrichment analysis of differential metabolites in petals

**Figure 8:**
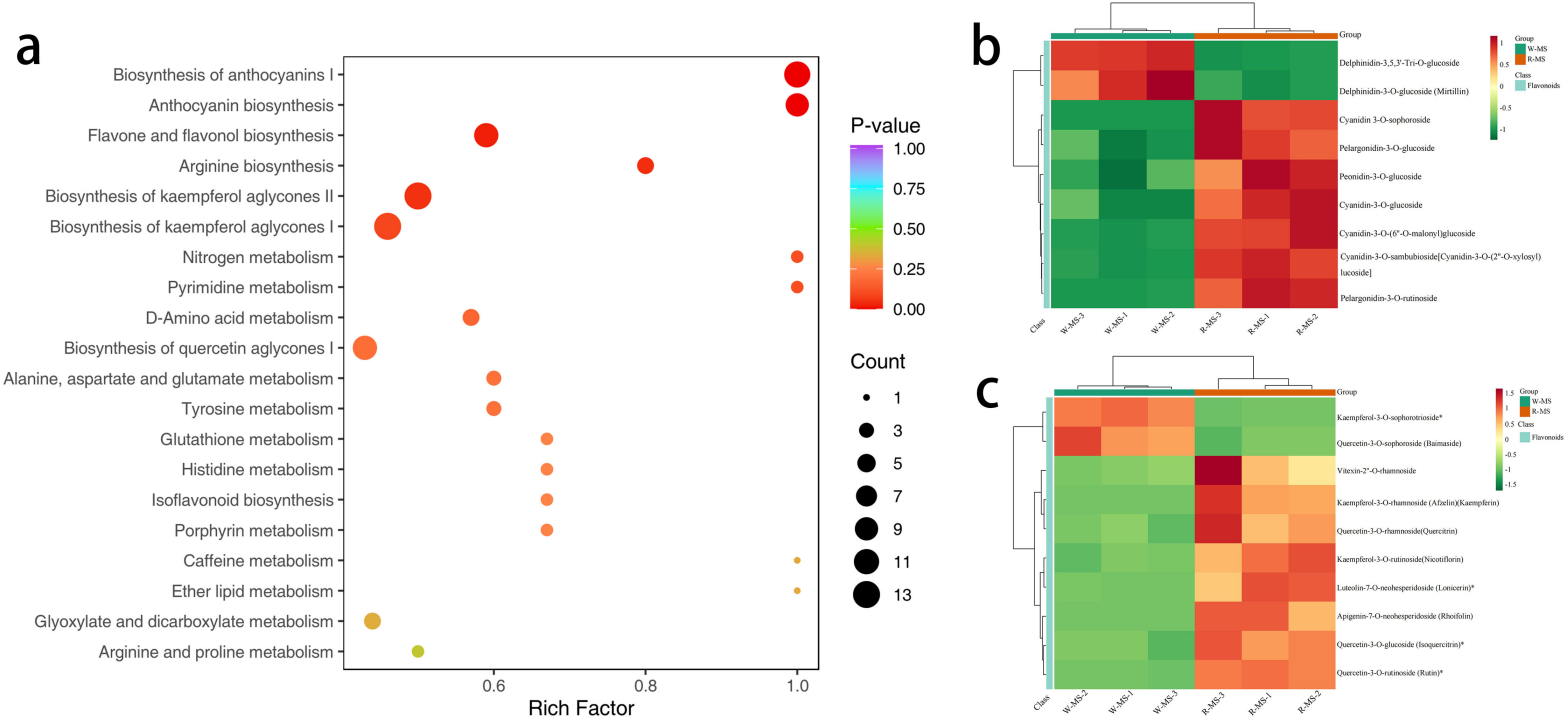
KEGG functional annotation and enrichment analysis of differential metabolites in petals. **(a)** The horizontal axis represents the Rich Factor for each pathway, and the vertical axis lists the pathway names (sorted by P-value). The color of the points reflects the P-value, with redder points indicating more significant enrichment, and the size of the points representing the number of enriched DMs. **(b, c)** The horizontal axis represents the sample names, and the vertical axis represents DMs. Different colors represent the different values obtained from normalized relative content (red indicates high content, green indicates low content). The annotation bar above the heatmap corresponds to the sample grouping (Group), and the dendrogram on the left of the heatmap represents the hierarchical clustering results of differential metabolites. The annotation bar to the right of the clustering tree corresponds to the primary classification of substances (Class), with different colors representing different substance categories.

KEGG pathway enrichment analysis was performed on the 419 DMs (**Figure 8a**), and the top 20 pathways ranked by P-value were displayed from smallest to largest. The results indicated that DMs related to the synthesis of anthocyanins and flavonoids were mainly concentrated in the Arginine biosynthesis (ko00942) and Flavone and flavonol biosynthesis (ko00944) pathways. Cluster analysis of the DMs in these two metabolic pathways revealed that in Arginine biosynthesis (**Figure 8b**), Delphinidin-3,5,3’-Tri-O-glucoside and Delphinidin-3-O-glucoside (Mirtillin) showed significantly increased content in W-MS, while they were significantly decreased in R-MS. Conversely, Cyanidin 3-O-sophoroside, Pelargonidin-3-O-glucoside, and Peonidin-3-O-glucoside showed significantly decreased content in W-MS and significantly increased in R-MS. In the Flavone and flavonol biosynthesis pathway (**Figure 8c**), Vitexin-2’’-O-rhamnoside, Kaempferol-3-O-rhamnoside (Afzelin) (Kaempferin), Quercetin-3-O-rhamnoside (Quercitrin), and Kaempferol-3-O-rutinoside (Nicotiflorin) showed significantly increased content in R-MS and significantly decreased in W-MS.

### 3.3 Joint analysis

#### 3.3.1 Joint analysis of transcriptome and volatile organic metabolome

**Figure 9:**
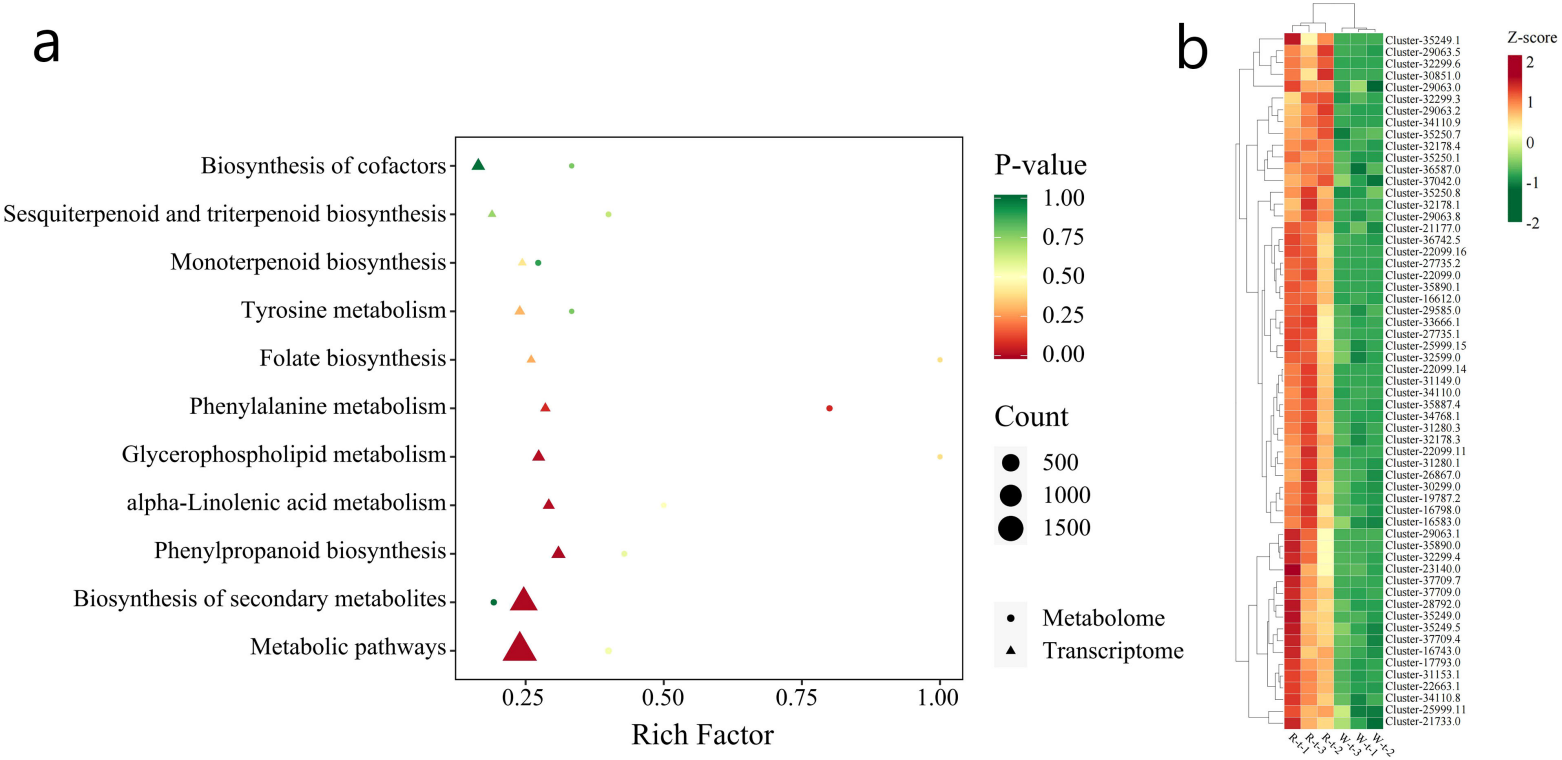
Jointanalysis of transcriptome sequencing and volatile metabolomie. **(a)** The horizontal axis represents the enrichment factor (Diff/Background) of the pathway in different omics, and the vertical axis represents the KEGG pathway name. The red-yellow-green gradient indicates the level of enrichment significance, represented by the p-value, The deeper the red color, the higher the abundance of metabolites and gene expression. The shape of the bubbles represents different omics, and the size of the bubbles represents the number of differential metabolites or genes, with larger points representing more substantial numbers. **(b)** The expression changes of 59 genes associated with Eugenol and Methyleugenol in *L.cernuum*.

In this study, the correlation between DEGs (Differentially Expressed Genes) and VOCs (Volatile Organic Compounds) was analyzed. By calculating the Pearson correlation coefficient between DEGs and VOCs, we selected those with an absolute correlation coefficient greater than 0.8 and a p-value less than 0.05. Then, based on the KEGG annotation results for genes and volatile metabolites, the relevant data were screened (**Figure 9a**). The genes and VOCs in the Phenylpropanoid biosynthesis (ko00940) and Phenylalanine metabolism (ko00360) pathways were selected for analysis. In these two pathways, when comparing R-GC and W-GC, the content of Eugenol and Methyleugenol in W-GC significantly decreased. There were 66 genes associated with these two metabolites, 59 of which were common to both Eugenol and Methyleugenol. Comparing these 59 genes with the PFam database, they mainly belong to three protein families: Peroxidase (13 genes), Aromatic amino acid lyase (10 genes), and AMP-binding enzyme (7 genes). Comparing with the Nr database, the 59 associated genes were mainly annotated to five gene families: Peroxidase Family (8 genes), Cinnamoyl-CoA Reductase Family (8 genes), 4-Coumarate–CoA Ligase Family (4 genes), Phenylalanine Ammonia-Lyase Family (3 genes), and Caffeoyl-CoA O-Methyltransferase Family (3 genes). All 59 associated genes in W-GC were down-regulated (**Figure 9b**).

#### 3.3.2 Joint analysis of the transcriptome and metabolome

**Figure 10:**
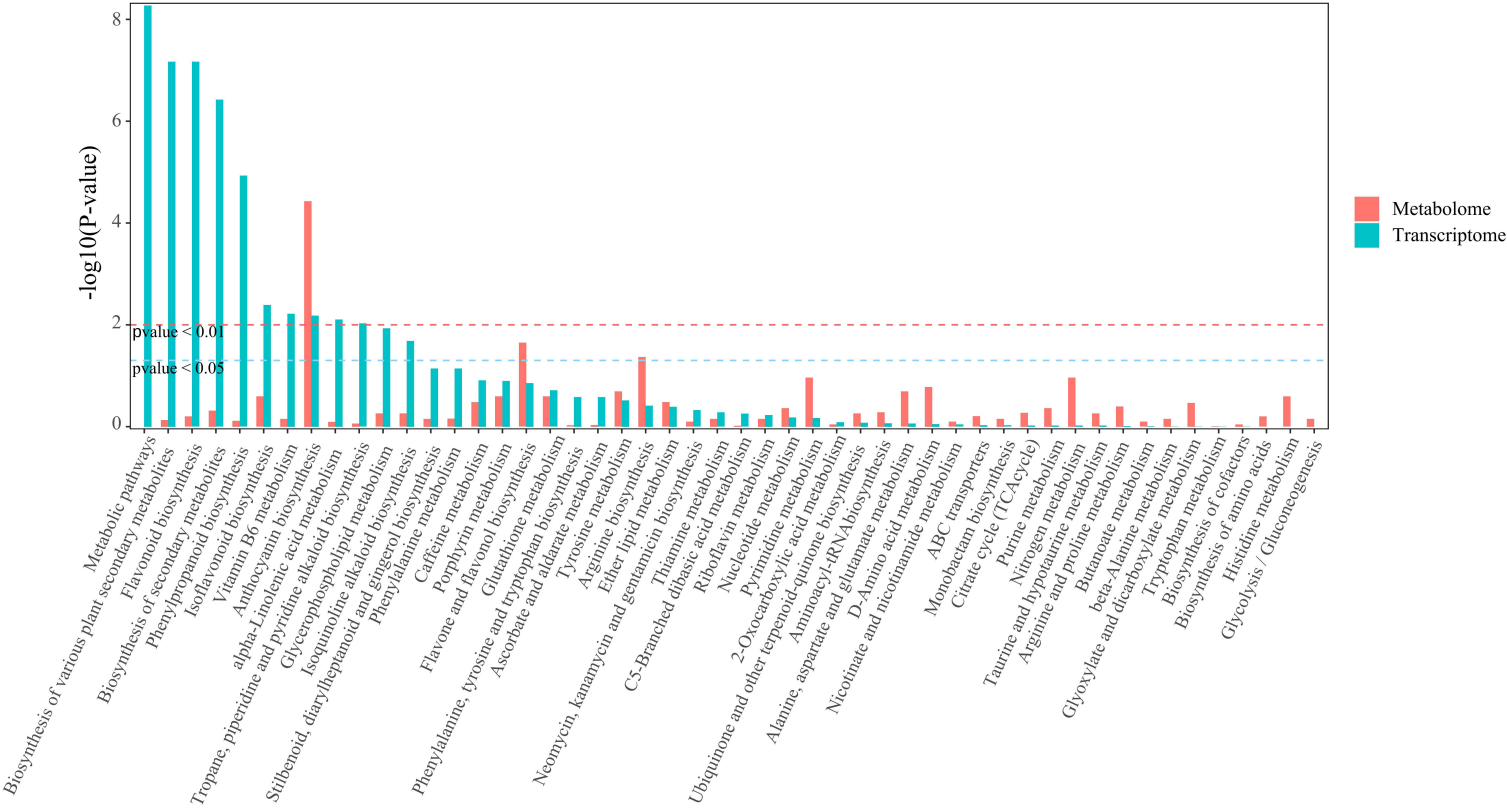
KEGG enrichment map of joint analysis of the transcriptome and metabolome.

The correlation between DEGs and DMs in L.cernuum was analyzed using their quantitative values. By calculating the Pearson correlation coefficient between DEGs and DMs, we selected those with an absolute correlation coefficient greater than 0.8 and a p-value less than 0.05. Then, based on the KEGG annotation results for genes and metabolites, relevant data were screened. As shown in **Figure 10**, the number of DEGs and DMs in the Anthocyanin biosynthesis (ko00942) pathway reached a highly significant level. Additionally, DEGs and DMs in the Phenylpropanoid biosynthesis (ko00940) and Flavonoid biosynthesis (ko00941) pathways were also selected for analysis.

When comparing R-MS and W-MS, in the Phenylpropanoid biosynthesis pathway, 37 genes and the metabolites Chlorogenic acid (3-O-Caffeoylquinic acid), Sinapinaldehyde, and p-Coumaric acid showed changes, with both gene expression and metabolite content being down-regulated in W-MS. These 37 genes belong to the Phenylpropanoid Pathway Enzymes, Carbohydrate Metabolism Enzymes, and Aldehyde Dehydrogenase Family categories, with the Phenylpropanoid Pathway Enzymes mainly including the Phenylalanine Ammonia Lyase (PAL), 4-Coumarate–CoA Ligase (4CL), and Cinnamoyl-CoA Reductase (CCR) families. It is worth noting that these 37 genes are also associated with the VOCs Eugenol and Methyleugenol.

In the Flavonoid biosynthesis pathway, a larger number of genes showed changes. A total of 71 genes and 7 metabolites, including Aromadendrin (Dihydrokaempferol), Catechin, Chlorogenic acid (3-O-Caffeoylquinic acid), Epicatechin, Hesperetin, Hesperetin-7-O-neohesperidoside (Neohesperidin), and Naringenin-7-O-glucoside (Prunin), showed changes. Compared to R-MS, 58 of the 71 genes in W-MS were down-regulated, and 13 were up-regulated. Among the 7 metabolites, the content of Aromadendrin and Hesperetin increased, while the other 5 metabolites decreased. These 71 genes mainly belong to the Flavonoid Biosynthesis Enzymes and Phenylpropanoid Pathway Enzymes categories, with the Flavonoid Biosynthesis Enzymes primarily including Chalcone Synthase (CHS), Chalcone Isomerase (CHI), Flavanone 3-Hydroxylase (F3H), Flavonol Synthase (FLS), Flavonoid 3’-Hydroxylase (F3’H), Dihydroflavonol 4-Reductase (DFR), Anthocyanidin Synthase (ANS), and Anthocyanidin Reductase (ANR).

In the Anthocyanin biosynthesis pathway, 7 genes and 9 metabolites showed changes. Compared to R-MS, 6 of the 7 genes in W-MS were down-regulated, and 2 were up-regulated. Among these 7 genes, 5 belonged to UDP-glucose: anthocyanidin 3-O-glucosyltransferase (UFGT), while 2 were unclassified. The 9 metabolites can be categorized into 4 types, with the content of Cyanidin, Pelargonidin, and Peonidin decreasing in W-MS, but the content of Delphinidin increasing.

The horizontal axis represents the KEGG pathway names, and the vertical axis represents the significance test p-value for enrichment in the pathway. Red and green indicate metabolomics and transcriptomics, respectively.

### 3.4 Validation of related genes

**Figure 11:**
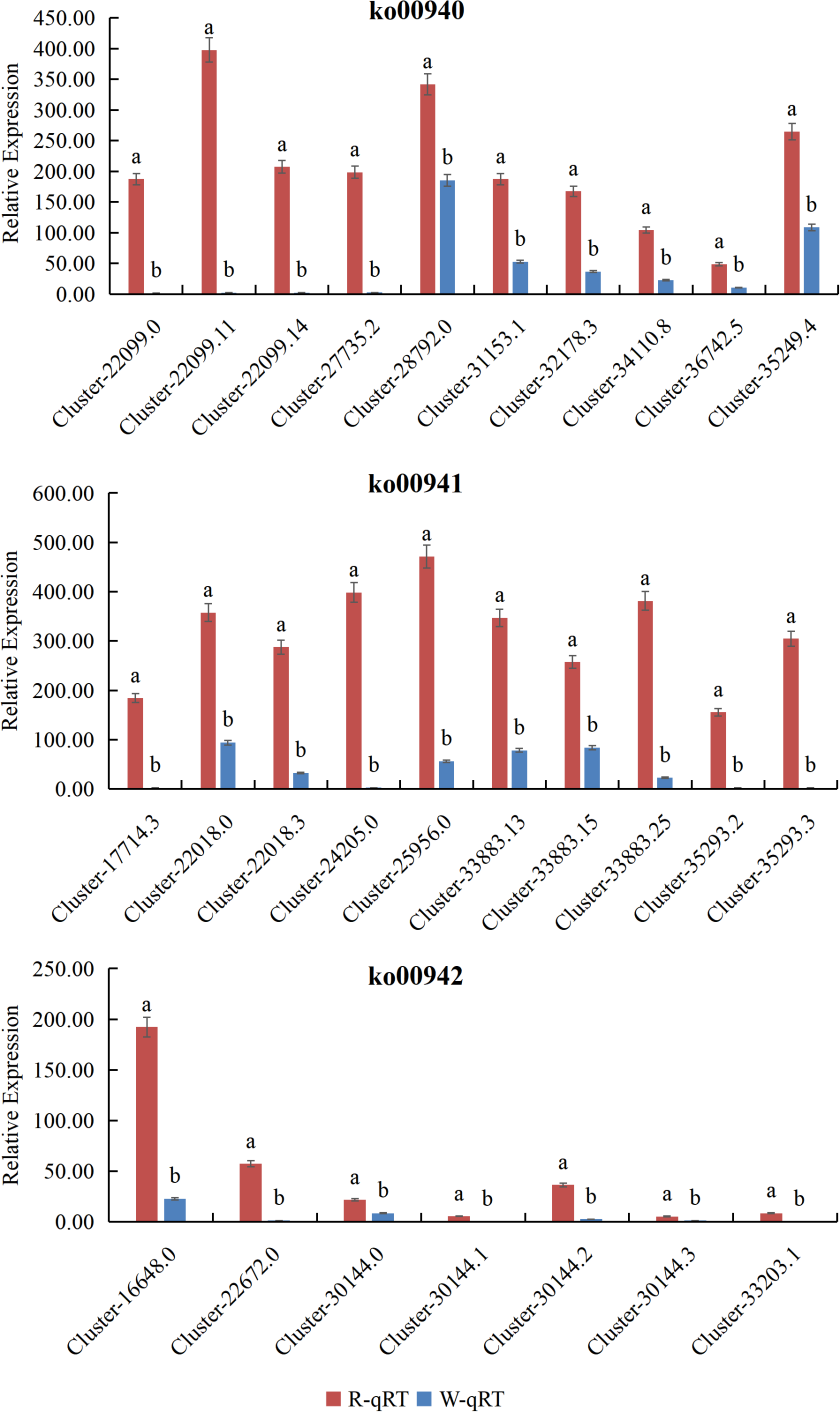
Real-time quantitative PCR validation of 27 DEGs.

To confirm the key DEGs involved in the Phenylpropanoid Pathway, Flavonoid biosynthesis, and Anthocyanin biosynthesis pathways, RNA sequencing results were used. The three most significantly differentially expressed DEGs from each pathway were selected for expression pattern analysis using real-time quantitative PCR. The results from qRT-PCR showed that the expression of 27 DEGs in *L. cernuum* and *L. cernuum* var. *album* was down-regulated, with a highly significant difference. These results indicate that not only is the expression trend of qRT-PCR data consistent with RNA-seq data, but they also validate the differential expression of these key genes in different samples (**Figure 11**).

Different lowercase letters represent significant differences.

## 4 Discussion

Research on the biosynthetic pathways of plant pigments and fragrances primarily focuses on the anthocyanin and flavonoid biosynthetic pathways (Liu et al., 2021), as well as the biosynthesis of terpenoids, phenylpropanoids, and fatty acid-derived VOCs (Picazo-Aragonés et al., 2020). In the biosynthetic pathway of plant pigments, anthocyanins and flavonoids are the primary pigments responsible for determining flower color (Enaru et al., 2021). The biosynthesis of anthocyanins and flavonoids begins with the conversion of phenylalanine to cinnamic acid by PAL, followed by the catalysis of chalcone formation from cinnamoyl-CoA and three molecules of malonyl-CoA by CHS. This is then converted into flavanone by CHI. Subsequently, flavanone undergoes a series of enzymatic reactions, including those catalyzed by DFR and ANS, ultimately producing anthocyanins. Other flavonoid derivatives are formed through enzymatic modifications such as hydroxylation, methylation, and glycosylation, contributing to the diversity and vividness of plant colors. The biosynthetic pathways of floral fragrances primarily involve the synthesis of terpenoids, phenylpropanoids, and fatty acid-derived compounds (Lv et al., 2024). Terpenoids are synthesized through the methylerythritol phosphate (MEP) pathway and the mevalonate (MVA) pathway (Bergman et al., 2024), with key enzymes including geranyl diphosphate synthase (GPPS) and terpene synthases (TPS). Phenylpropanoid compounds are derived from cinnamic acid, produced by PAL, and are further synthesized into aromatic substances such as eugenol and vanillin. Fatty acid derivatives are synthesized through the lipoxygenase (LOX) pathway (Viswanath et al., 2020), producing green leaf volatiles (GLVs) such as cis-3-hexenal and cis-3-hexenol. These pathways collectively generate a complex array of floral fragrance compounds, contributing to the distinctive scents of plants.

**Figure 12:**
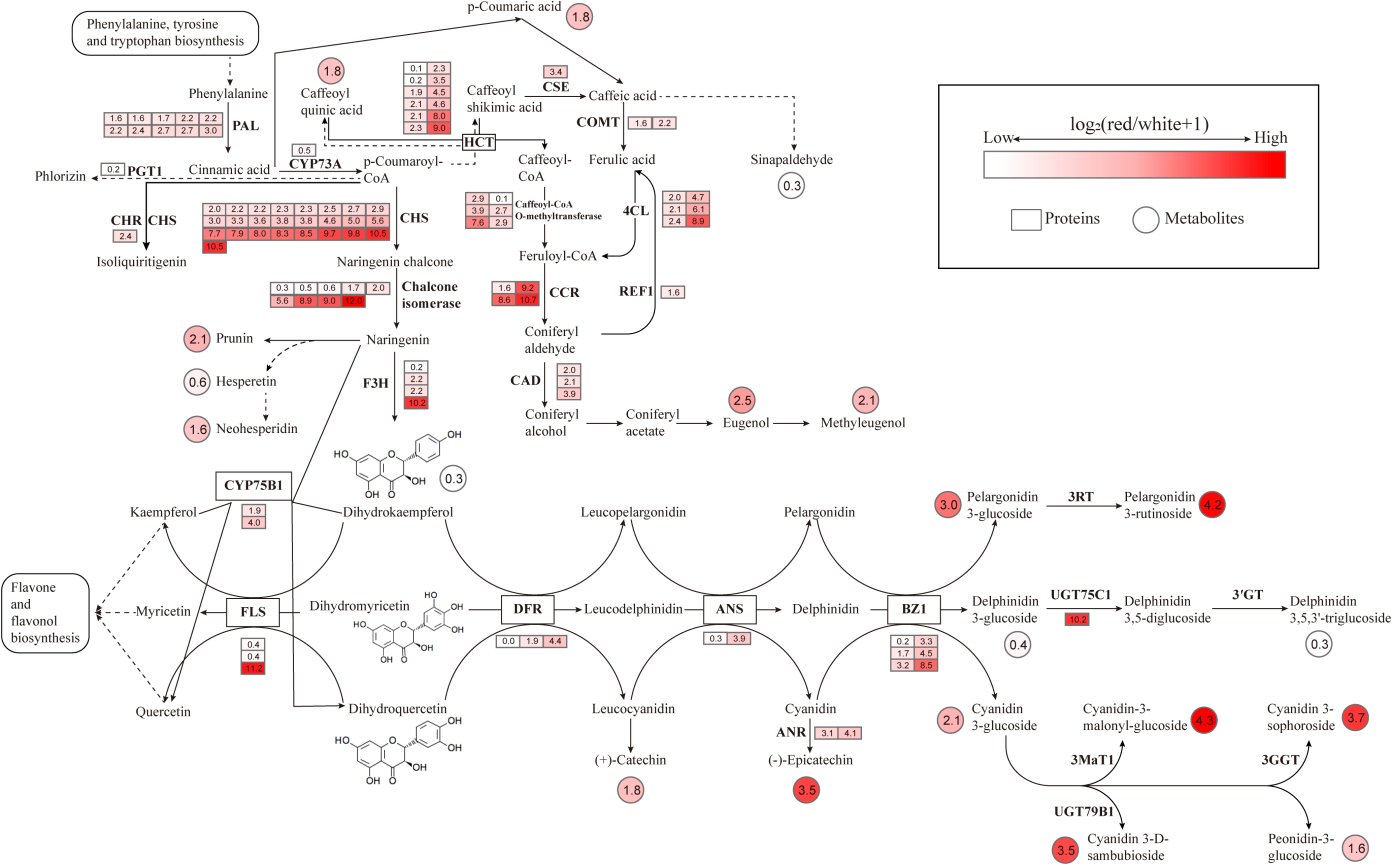
Integrated analysis of transcriptomic and metabolomic data in the biosynthesis pathways of volatile metabolites, flavonoids, and anthocyanins circles represent metabolites, squares represent predicted proteins, and the color intensity reflects the abundance of metabolites and gene expression.

To better understand the relationship between gene expression and the production of volatile metabolites, flavonoids, and anthocyanins, transcriptomic and metabolomic data were mapped onto the Phenylpropanoid Pathway, Flavonoid Biosynthesis, and Anthocyanin Biosynthesis pathways (**Figure 12**).

Through the joint analysis of transcriptomics, metabolomics, and volatolomics, it was found that compared to *L. cernuum* var. *album*, *L. cernuum* had higher contents of flavonoids, anthocyanins, and volatile organic compounds related to floral fragrance and sweetness. The Phenylpropanoid Pathway plays a crucial role not only in plant resistance and lignin biosynthesis but also in the formation and variation of flower color and fragrance. Changes in this pathway directly affect the synthesis of precursors for anthocyanins and volatile metabolites (Feng et al., 2021; Khan et al., 2022). p-Coumaric acid is a key intermediate in the Phenylpropanoid Pathway. Phenylalanine, under the action of PAL, the initial enzyme in the phenylpropanoid metabolism pathway, is deaminated and converted to Cinnamic acid, which is then transformed into p-Coumaric acid by C4H. 4CL further converts p-Coumaric acid into p-Coumaroyl-CoA. This intermediate is then subjected to a series of enzymatic reactions—demethylation, reduction, and methylation—to ultimately produce Eugenol, which subsequently leads to the formation of Methyleugenol (Kumari et al., 2017). Both of these metabolites are essential components of lily fragrance (Wei et al., 2022) and are also major volatile active substances in many aromatic plants and flowers (Ghissing and Mitra, 2022). For instance, the fragrance of *Ocimum basilicum* is attributed to Eugenol, Eugenol and Methyleugenol are also the primary components of the fragrance in (Shah et al., 2022) *Lonicera japonica* (Li et al., 2022), *Tillandsia* (Lo et al., 2021) and *Rosa hybrid* (Feng et al., 2022). In different color groups of Chimonanthus praecox, Eugenol is one of the main components of the fragrance, with significant variation in content across different flower colors, thereby impacting the floral scent characteristics. Eugenol also interacts with other fragrance components, imparting each flower color of *C.praecox* with unique aromatic features (Meng et al., 2021). In this study, the reduced fragrance and color change to pure white in *L. cernuum* var. *album* were linked to the downregulation of key genes (such as PAL, 4CL, and CCR) in the Phenylpropanoid Pathway. This downregulation led to a significant decrease in the levels of volatile organic compounds like Eugenol and Methyleugenol, as well as pigments like anthocyanins and flavonoids, thus affecting both fragrance and flower color. The reduction in p-Coumaric acid, a crucial intermediate in this pathway, directly impacted the synthesis of precursor compounds for fragrance and color, contributing to the diminished scent and altered color of *L. cernuum* var. *album*.

p-Coumaric acid also serves as a key precursor in the biosynthesis pathways of anthocyanins and flavonoids. It is first catalyzed by 4CL to form p-Coumaroyl-CoA, which then undergoes a condensation reaction with three molecules of malonyl-CoA under the action of CHS to form Chalcone. Chalcone is subsequently isomerized into flavanone by CHI. Flavanone is then converted into dihydrokaempferol via a hydroxylation reaction catalyzed by F3H. Dihydrokaempferol is further reduced to leucoanthocyanidin by DFR, which is then oxidized into Anthocyanidin by ANS. Finally, anthocyanidin is glycosylated by UFGT to form a stable anthocyanin, cyanidin-3-glucoside. Through these enzymatic reactions, p-Coumaric acid connects Flavonoid Biosynthesis to the biosynthesis of anthocyanins and flavonoids, directly influencing flower color variation in plants (Fatihah et al., 2022).

In the comparison between *L. cernuum* and *L. cernuum* var. *album*, five metabolites—Catechin, Chlorogenic acid, Epicatechin, Neohesperidin, and Prunin—were found to decrease in the Flavonoid Biosynthesis pathway in *L. cernuum* var. *album*. These metabolites are closely related to color changes, with Catechin and Epicatechin being intermediates in the anthocyanin synthesis pathway that directly affect anthocyanin accumulation and color expression (Xiong et al., 2013). Chlorogenic acid not only acts as a precursor in anthocyanin synthesis but also may regulate color stability through its antioxidant properties (Ye et al., 2019). Neohesperidin and Prunin participate in the synthesis of flavonoids and flavonols, influencing the hue and intensity of flower color (Zhou et al., 2022). Correspondingly, 58 genes were downregulated in the Flavonoid Biosynthesis pathway, including key genes involved in the entire anthocyanin and flavonoid synthesis process, such as CHS, CHI, F3H, and FLS. The downregulation of these genes directly resulted in a reduced synthesis of anthocyanins and flavonoids, further impacting the Anthocyanin Biosynthesis pathway. “Through the study of flower development stages in white-flowered Lilium longiflorum, the results showed that the absence of two structural genes, F3H and DFR, as well as the bHLH2 transcription factor, led to the inability or insufficient synthesis of anthocyanins. This is similar to the findings of this study (Fatihah et al., 2022).

In the Anthocyanin Biosynthesis pathway, the levels of three metabolites—Cyanidin, Pelargonidin, and Peonidin—were found to decrease. These metabolites play crucial roles in the coloration of flowers, with Cyanidin associated with red and purple, Pelargonidin with orange or red, and Peonidin with purple-red (Wang et al., 2023). The reduction in these metabolites directly led to the transformation of the purple-red flowers of *L. cernuum* into white. These metabolites are substrates of UFGT, and in the Anthocyanin Biosynthesis pathway, five out of seven downregulated genes belong to UFGT, indicating that the glycosylation process of anthocyanins was inhibited. This inhibition likely caused anthocyanins to degrade or convert into other colorless compounds, preventing their accumulation in petal cell vacuoles, leading to the weakening or loss of color (Wu et al., 2017; Muhammad et al., 2022). Although Delphinidin levels increased in this pathway, it did not exhibit color, possibly due to its poor thermal stability. Some studies suggest that an increased number of substituents on the β-ring reduces thermal stability. Delphinidin contains three hydroxyl substituents and is considered less stable (Sinela et al., 2017), while Pelargonidin, with only one hydroxyl substituent, is regarded as the most stable aglycone (Fleschhut et al., 2006). In the study of Lilium chinense var. rubrum, it was found that the high expression level of the UFGT gene in pink leaves was consistent with the stable formation of anthocyanins. The UFGT gene directly participates in the stable synthesis process of anthocyanins (Zhang et al., 2022).

## 5 Conclusion

This study comprehensively analyzed the transcriptomic, metabolomic, and volatolomic data of *L. cernuum* var. *album* and *L. cernuum*, revealing the molecular mechanisms underlying the coordinated changes in flower color and fragrance. The experimental results indicate that the pure white color and subtle fragrance of *L. cernuum* var. *album* are primarily attributed to the low expression of key genes in the Phenylpropanoid Pathway and the reduction in related metabolites. Metabolomic analysis showed that the flavonoid and anthocyanin content in *L. cernuum* var. *album* was significantly lower than in *L. cernuum*. This difference is driven by the downregulation of multiple key genes in the Phenylpropanoid Pathway, including PAL, C4H, and 4CL, which led to decreased accumulation of p-Coumaric acid, subsequently affecting the synthesis of downstream anthocyanins and volatile organic compounds. In terms of volatile metabolites, the contents of Eugenol and Methyleugenol were significantly lower in *L. cernuum* var. *album* compared to *L. cernuum*, indicating a weakening or alteration of fragrance. This reduction in volatile organic compounds is closely related to the downregulated expression of genes in the Phenylpropanoid Pathway.

Moreover, in *L. cernuum* var. *album*, the decreased content of metabolites such as Catechin, Chlorogenic acid, Epicatechin, Neohesperidin, and Prunin in the Flavonoid Biosynthesis pathway also contributed to the weakening of flower color. Specifically, the reduced levels of the intermediate metabolites Catechin and Epicatechin in the anthocyanin synthesis pathway likely directly impacted anthocyanin accumulation and color expression. Gene expression data further support this view, as 58 genes involved in the Flavonoid Biosynthesis pathway were downregulated in *L. cernuum* var. *album*, affecting the entire process of anthocyanin and flavonoid synthesis. The downregulation of these genes led to a reduction in the synthesis of anthocyanins and flavonoids, which in turn affected flower color. In the Anthocyanin Biosynthesis pathway, the contents of Cyanidin, Pelargonidin, and Peonidin were significantly reduced in *L. cernuum* var. *album*, directly explaining the color change from purple-red to white. Particularly, the downregulation of the UFGT gene inhibited the glycosylation process of anthocyanin molecules, leading to their degradation or conversion into other colorless compounds.

In summary, this study elucidated the reasons behind the differences in flower color and fragrance between *L. cernuum* var. *album* and *L. cernuum* through detailed analysis of gene expression and metabolite changes. The expression levels of key genes in the Phenylpropanoid Pathway determined the synthesis of p-Coumaric acid and its downstream metabolites, thereby influencing the coordinated changes in flower color and fragrance. These findings provide an important theoretical foundation for further understanding the molecular regulatory mechanisms of flower color and scent and offer valuable insights for the breeding and improvement of superior lily cultivars.

## Data Availability

The original contributions presented in the study are included in the article/supplementary material, Further inquiries can be directed to the corresponding author/s.
